# Activity-Guided Isolation of *sn*-1,3-Dipalmitoyl-2-oleoylglycerol as Protein Tyrosine Phosphatase 1B and α-Glucosidase Inhibitor from Skipjack Tuna (*Katsuwonus pelamis*)

**DOI:** 10.3390/ijms27156914

**Published:** 2026-08-01

**Authors:** Md Yousof Ali, Da Hye Kim, Hee Jin Jung, Taek Jeong Nam, Jae Sue Choi

**Affiliations:** 1 Department of Clinical Neurosciences, Hotchkiss Brain Institute and Alberta Children’s Hospital Research Institute, Cumming School of Medicine, University of Calgary, Calgary, AB T2N 4N1, Canada; mdyousof.ali@ucalgary.ca; 2 Department of Food Science and Nutrition, Pukyong National University, Busan 48513, Republic of Korea; md2253@naver.com (D.H.K.); king2046@hanmail.net (H.J.J.); namtj@pknu.ac.kr (T.J.N.)

**Keywords:** *Katsuwonus pelamis*, Skipjack tuna heart, anti-diabetic, *sn*-1,3-dipalmitoyl-2-oleoylglycerol, PTP1B

## Abstract

The skipjack tuna (*Katsuwonus pelamis*) is one of the least commercially exploited tuna globally. The possible medicinal benefits of *K. pelamis* extract have not been explored. We investigated the 70% ethanol (EtOH) soluble fractions of skipjack tuna heart for the inhibition of protein-tyrosine phosphatase 1B (PTP1B) and α-glucosidase. The dichloromethane (CH_2_Cl_2_) fraction significantly inhibited the activities of PTP1B and α-glucosidase. Repeated column chromatography of the active CH_2_Cl_2_ fraction based on bioactivity-guided fractionation yielded cholesterol, cholesteryl myristate, and *sn*-1,3-dipalmitoyl-2-oleoylglycerol; the latter significantly inhibited PTP1B and α-glucosidase. Kinetic study revealed that *sn*-1,3-dipalmitoyl-2-oleoylglycerol showed mixed-type inhibition against PTP1B. Docking simulations showed that *sn*-1,3-dipalmitoyl-2-oleoylglycerol selectively inhibited PTP1B and α-glucosidase by targeting its active site and exhibited good binding affinity, with a docking score of −7.4 and −7.1 kcal/mol, respectively. Moreover, 70% EtOH, CH_2_Cl_2_ and EtOAc fractions, and *sn*-1,3-dipalmitoyl-2-oleoyl glycerol significantly inhibited ONOO−mediated albumin nitration. These results implicate tuna heart extract as a potential functional food ingredient for the prevention and treatment of diabetes and its associated complications.

## 1. Introduction

Diabetes mellitus (DM) is a chronic metabolic disorder, which is increasing in prevalence worldwide [1]. DM is characterized by high concentrations of blood sugar. This can cause serious long-term complications. Treatment mostly focuses on reducing fluctuations in blood sugar and attenuating subsequent complications [2]. DM remains incurable. Insulin therapy is beneficial as a treatment of DM, but drawbacks include insulin resistance and development of fatty liver upon prolonged insulin use [3]. Antioxidants and inhibitors of protein-tyrosine phosphatase 1B (PTP1B) and α-glucosidase could be effective adjunct therapeutic options to attenuate the noxious effects of glucose. Inhibition of α-glucosidase at the intestinal brush border of the intestine may play a role in lowering postprandial hyperglycemia [4,5]. Furthermore, PTP1B inhibitors can be used to modulate insulin receptor phosphorylation and insulin therapy can reduce hyperglycemia and maintain normoglycemia [6,7]. Therefore, the use of agents or substances that reduce postprandial hyperglycemic may be useful for the treatment of diabetes and diabetes-associated complications. Furthermore, the action of ONOO− produces nitrotyrosine, and the presence of nitrotyrosine residues can be used to indirectly infer the formation of ONOO− [8]. The plasma of diabetic patients confirms that nitrotyrosine levels rise as a result of tyrosine nitration by ONOO−, which frequently results in a loss of protein activity [9]. However, nitrotyrosine synthesis has also been reported to reduce acute glycemia and hyperglycemia [10]. PTP1B, α-glucosidase, and nitrotyrosine are therefore interesting candidates for the discovery of novel treatments for DM and other associated metabolic disorders.

Skipjack tuna (*Katsuwonus pelamis*) is one of the most important commercial fish species distributed in both offshore waters and open seas in tropical and temperate regions around the world, including the Pacific, Atlantic, and Indian Oceans [11]. Skipjack tuna catch increased from 1,381,070 tons in 2010 to 1,952,138 tons in 2014. The meat is typically canned, with about one-third of the whole fish not utilized. Tuna byproducts are primarily used to produce low market-value products, such as fishmeal and fertilizer, because of their off-flavors and poor functional properties [12]. But recovery and conversion of tuna byproducts to value-added products is an option. Parts of the tuna head, including the tongue, cheeks, nape, heart, and upper head, can be consumed as a meat source. The heart and cheeks are considered by some consumers to be delicacies due to their unique taste and excellent texture. Additionally, fish byproducts can be enriched with bioactive protein, lipid, vitamins, and minerals from muscle protein, collagen, peptides, gelatin, oil, bone, and internal organs that remain after processing [13,14].

Tuna fish and its components have been demonstrated to possess a wide range of biological activities including anti-diabetic [15], antioxidant and antihypertensive [15,16,17], antiproliferative [18], antianemic [19], antimicrobial properties [20], lipid peroxidation [21], and monoamine oxidase inhibitory activities [22]. Very recently, we reported that 70% ethanol (EtOH) extract of tuna heart has anti-diabetic and antioxidant activities [15]. Here, we have extended these observations by investigating the PTP1B and α-glucosidase inhibitory capability of 70% EtOH soluble fractions of tuna heart in vitro. The inhibitory activities of three isolated compounds were evaluated against PTP1B and α-glucosidase. Kinetic analyses of the most active compound, *sn*-1,3-dipalmitoyl-2-oleoylglycerol, were performed by using Dixon and Lineweaver–Burk plots to determine the type of enzymatic inhibition. The interactions between *sn*-1,3-dipalmitoyl-2-oleoylglycerol and PTP1B and α-glucosidase were simulated using molecular docking, in which docking energy and PTP1B and α-glucosidase inhibition were examined. Additionally, we investigated the activity of 70% EtOH soluble fractions of skipjack tuna heart against ONOO−-mediated nitrated albumin. 

## 2. Results and Discussion

### 2.1. Isolation and Purification of Target Compounds

**Figure 1:**
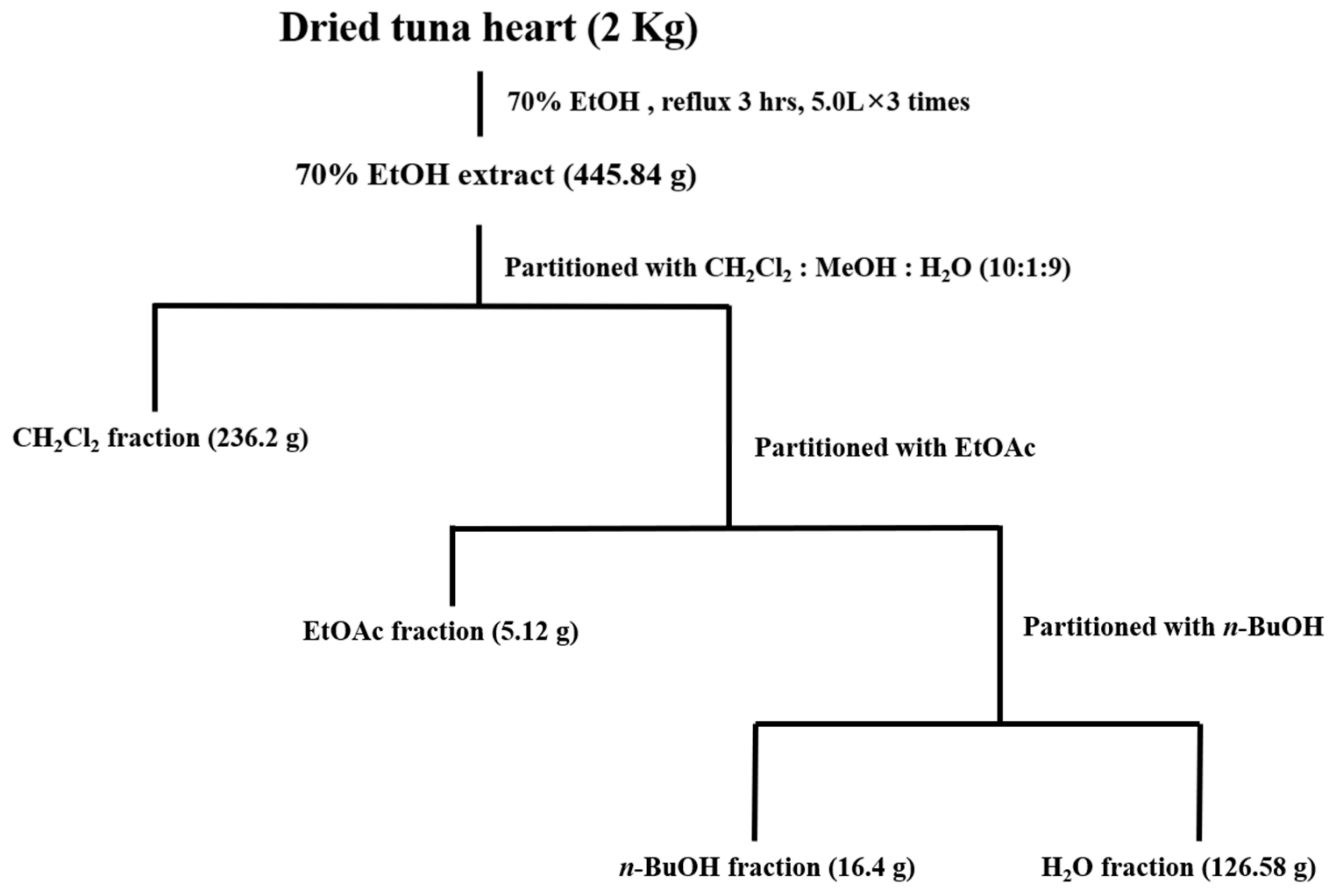
Scheme for extraction and fractionation of tuna heart extracts.

The extract (445.84 g) was suspended in distilled water and partitioned with dichloromethane (CH_2_Cl_2_), ethyl acetate (EtOAc), and *n*-butanol (*n*-BuOH) to yield the CH_2_Cl_2_ (236.2 g), EtOAc (5.12 g), and *n*-BuOH (16.4 g) fractions, respectively, as well as an aqueous residue (126.58 g) (Figure 1). Of the various fractions obtained from EtOH extracts of tuna fish heart, the anti-diabetic activity of the CH_2_Cl_2_ fraction from tuna heart was evaluated based on the higher inhibitory activity against PTP1B and α-glucosidase. This fraction most potently inhibited PTP1B and α-glucosidase (Table 1). The fraction was subjected to Si-gel column chromatography to isolate the bioactive compounds, which resulted in ten subfractions (F-1 to F-10), and they were further evaluated for PTP1B and α-glucosidase inhibitory activities. F-3 had the highest inhibition percentage among the tested subfractions and F-4 displayed high yield with significant inhibition of PTP1B and α-glucosidase activities (Table 2). Both fractions were used for further Si-gel chromatography, which revealed F3 and F4 to be cholesterol, cholesteryl myristate, and *sn*-1,3-dipalmitoyl-2-oleoylglycerol respectively.

### 2.2. Identification of Compounds

**Figure 2:**
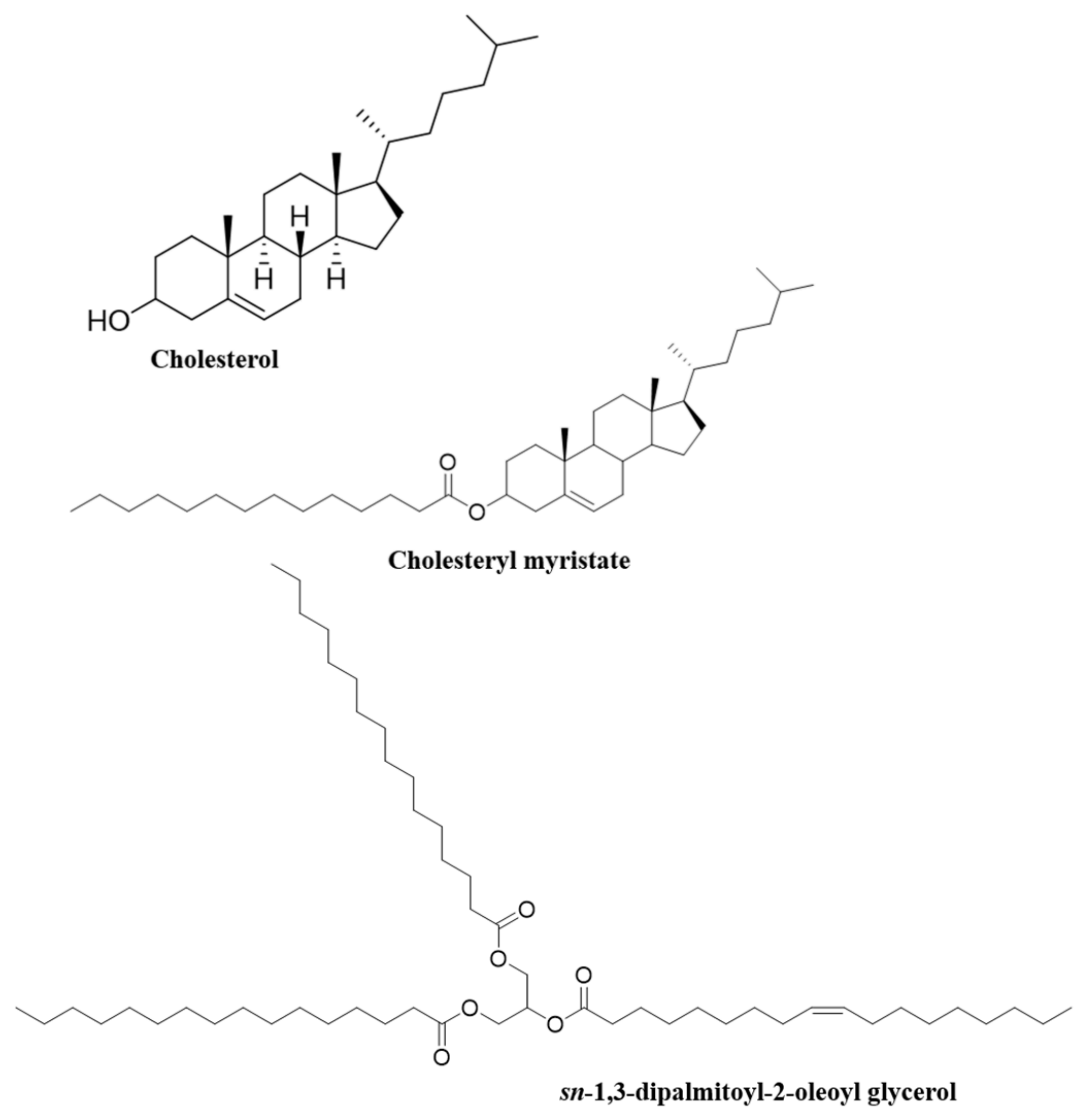
Chemical structures of the compounds isolated from tuna heart.

The structures of isolated compounds were identified by elucidating the one-dimensional NMR, 2D NMR and electrospray ionization-mass spectrometry (ESI-MS) data, and by comparing these data with those in references when available. Three compounds were identified from CH_2_Cl_2_ fraction of tuna heart extract: cholesterol [23], *sn*-1,3-dipalmitoyl-2-oleoylglycerol [24], and cholesteryl myristate [25]. Compound *sn*-1,3-palmitoyl-2-oleoylglycerol was obtained as a white amorphous solid, and its positive high resolution (HR)-ESI-MS showed a [M + Na]^+^ ion at *m*/*z* 855.7412, confirming a molecular formula of C_53_H_100_O_6_. The fatty acids esterified to the glycerol in the triglycerides were determined to be oleic acid and two palmitic acids from the HR-EI-MS spectra of hydrolysis products of triglyceride [26] and ^1^H NMR integrations of the resonances at δ 0.87 (3H, t, *J* = 6.9 Hz, CH_3_), 2.00 (allylic, CH_2_), and 5.34, 5.31 (m, olefinic H) for oleic acid, and δ 0.87 (3H, t, *J* = 6.9 Hz, CH_3_) for palmitic acid. The position of esterified fatty acid was determined by comparison with the data of Simova et al. [24]. The ^13^C NMR spectrum showed signals at δ 173.35 and 172.93 for the ester bond between glycerol and fatty acids, respectively; δ 130.02 and 129.71 for the double bond in oleic acid; and δ 62.11 and 68.84 for glyceryl CH_2_ and glyceryl CH, respectively. The ^1^H NMR spectrum also indicated unsubstituted proton signals at δ 5.34 and 5.31 (m, olefinic H), which correlated with the double bond in oleic acid, and δ 4.30 (2H, dd, *J* = 4.4, 11.92 Hz), 4.15 (2H, dd, *J* = 5.9, 12.08 Hz), 5.29 (1H, m), which correlated with glyceryl CH_2_ and glyceryl CH. In addition, a methyl signal at δ 0.87 (3H, t) was observed. Thus, the structure was elucidated as *sn*-1,3-palmitoyl-2-oleoylglycerol. The structures of the isolated compounds are shown in Figure 2.

### 2.3. Inhibition of PTP1B

**Figure 3:**
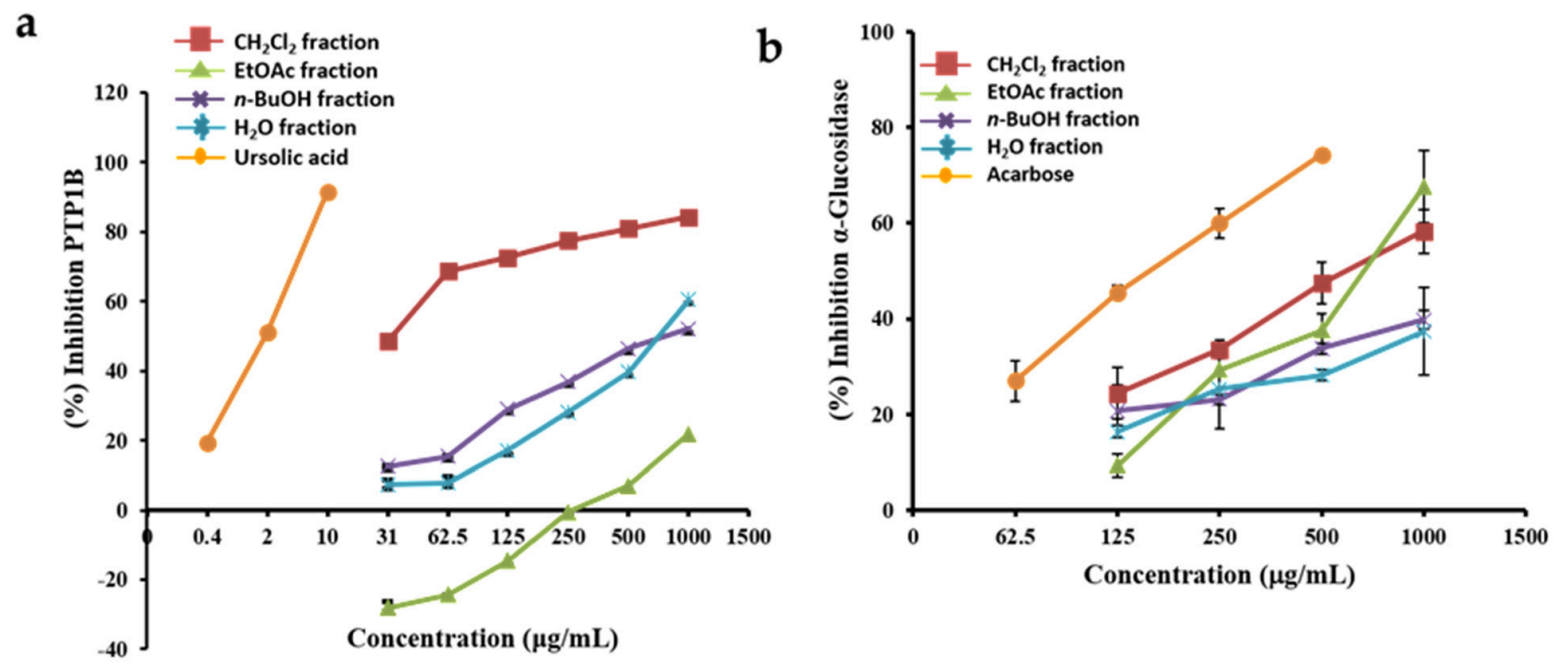
Effects of tuna heart extract on protein tyrosine phosphatase 1B activity (**a**) and α-glucosidase activity (**b**). A concentration range of 30–1000 µg/mL was observed. Ursolic acid and acarbose were used as positive controls in respective assays. All values are expressed as the mean ± SEM of triplicate experiments.

PTP1B is a major nontransmembrane phosphotyrosine phosphatase that is expressed ubiquitously in classical insulin-targeted tissues. PTP1B acts as a negative regulator of the insulin signaling pathway and is one of the most attractive molecular targets for the treatment of DM [7,27]. Previous independent studies revealed that PTP1B knock-out mice processed lower insulin and glucose levels with increased sensitivity to insulin, and resistance to high-fat induced weight gain without any adverse effects [28,29], which implicated PTP1B as a potential target for the treatment of type II DM and obesity. Development of PTP1B inhibitors as potential therapeutic compounds for type II DM has been intensively explored, but these efforts have been hindered by low selectivity compared to other PTPs and poor cell permeability [30]. Some PTP1B inhibitors are undergoing clinical trials [31]. In the present study we investigated the PTP1B inhibitory potential of tuna heart extract (Figure 3a and Table 1). Among the different tuna heart fractions, the CH_2_Cl_2_ fraction showed superior inhibitory activity against PTP1B with an IC_50_ value of 33.53 ± 0.33 µg/mL, as compared to the positive control, ursolic acid (IC_50_ 4.95 ± 0.05 µg/mL). The *n*-BuOH and H_2_O fractions exhibited weak inhibitory activity against PTP1B with IC_50_ values of 884.73 ± 13.11 and 758.99 ± 47.39 µg/mL respectively, while the EtOAc fraction did not show inhibitory activity within the tested concentrations. In order to evaluate the PTP1B inhibitory activity of the three isolated compounds from tuna heart extract, the inhibitory potential of PTP1B was evaluated using *p*-NPP as substrate. The results, expressed as IC_50_ values, are presented in Table 3. Among the tested compounds, *sn*-1,3-dipalmitoyl-2-oleoyl glycerol displayed significant inhibitory activity against PTP1B with an IC_50_ value of 31.01 ± 0.39 µM, while cholesteryl myristate and cholesterol showed weak inhibitory activity with IC_50_ values of 335.57 ± 1.24 and 518.13 ± 2.56 µM, respectively. *Sn*-1,3-dipalmitoyl-2-oleoyl glycerol was determined to be composed of three components: glycerol, palmitic acid, and oleic acid. As shown in Table 4, oleic acid exhibited significant inhibitory activity against PTP1B with an IC_50_ value of 64.19 ± 0.22 µM, whereas palmitic acid showed moderate activity with an IC_50_ value of 204.58 ± 15.05 µM, and glycerol did not show inhibitory activity within the tested concentration. Despite the identification of many potent natural and synthetic compounds [32,33,34,35,36,37], a PTP1B-inhibiting drug has yet to reach the clinic, because most of the PTP1B inhibitors discovered thus far are highly charged molecules and lack efficacy in vivo because they have weak oral bioavailability, poor membrane permeability and weak selectivity. Therefore, the purpose of this study was to identify new PTP1B inhibitors from skipjack tuna (*K. pelamis*) for the treatment of diabetes. To the best of our knowledge, this is the first report to describe the PTP1B inhibitory activity of *sn*-1,3-dipalmitoyl-2-oleoyl glycerol.

### 2.4. Inhibition of α-Glucosidase

Postprandial hyperglycemia is a major symptom in patients suffering from DM. Inhibitors of α-glucosidase can efficiently decrease postprandial hyperglycemia and help in the management of diabetes and related complications [4,38]. α-Glucosidase is a constituent of human intestinal cells at the brush border surfaces. Since the human intestine can only absorb monosaccharides, the hydrolysis of carbohydrates into glucose monomers by α-glucosidase is essential [39]. The hydrolysis is vital in digestion of carbohydrates and processing of glycoproteins. Therefore, α-glucosidase inhibitors have been recommended as a first-line therapy by the American Association of Clinical Endocrinologists and International Diabetes Federation due to their characteristics of safety, efficacy, lack of hypoglycemia, and cardiovascular tolerability [40,41]. However, synthetic α-glucosidase inhibitors have many undesirable adverse effects [42]. Many efforts have searched for effective, non-toxic, and inexpensive α-glucosidase inhibitors from natural resources that can prevent the risk of type 2 DM and related complications. The α-glucosidase inhibition potential of the tuna extract was measured (Figure 3b and Table 1). Among the different tuna heart fractions, the CH_2_Cl_2_ and EtOAc fractions showed significant inhibitory activity toward α-glucosidase with an IC_50_ value of 609.51 ± 20.89 and 612.55 ± 23.38 µg/mL, respectively, as compared to the positive control, acarbose (IC_50_ 170.02 ± 1.64 µg/mL). The *n*-BuOH and H_2_O fractions did not show inhibitory activity within the tested concentration. In addition, the isolated compounds *sn*-1,3-dipalmitoyl-2-oleoyl glycerol exhibited significant inhibitory activity against α-glucosidase with an IC_50_ value of 163.31 ± 1.15 µM, whereas cholesteryl myristate and cholesterol showed weak inhibitory activity with IC_50_ values of 419.46 ± 2.10 and 647.67 ± 3.64 µM, respectively (Table 3). 

Different classes of natural and synthetic α-glucosidase inhibitors have been reported [32,33,43,44,45], and among them, acarbose and voglibose can effectively control blood glucose levels after food intake and have been used clinically in the treatment of DM [46]. Acarbose is typically administered in combination with other anti-diabetic agents in a polypharmacological approach but is associated with severe side effects such as abdominal pain, flatulence, and diarrhea [47,48]. Therefore, it is necessary to search for alternatives that possess α-glucosidase inhibitory activity but without unacceptable side effects. In the present study, we found that the inhibitory potential of skipjack tuna-derived compound *sn*-1,3-dipalmitoyl-2-oleoylglycerol against α-glucosidase was much stronger than that of acarbose, a clinically used α-glucosidase inhibitor. Therefore, *sn*-1,3-dipalmitoyl-2-oleoylglycerol might be effective in controlling postprandial hyperglycemia by delaying the breakdown of polysaccharides.

### 2.5. Kinetic Parameters Studies of sn-1,3-Dipalmitoyl-2-oleoyl Glycerol Against PTP1B

**Figure 4:**
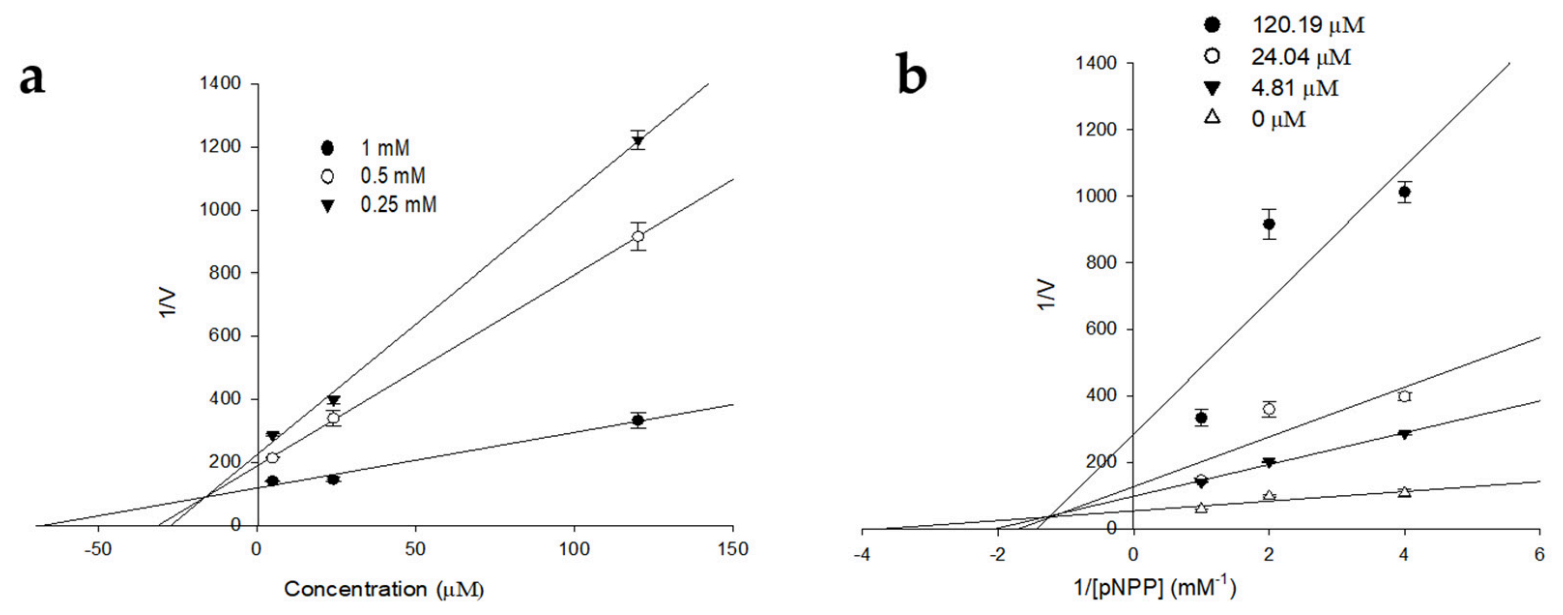
(**a**) Dixon plots of protein tyrosine phosphatases 1B (PTP1B) inhibition by *sn*-1,3-dipalmitoyl-2-oleoyl glycerol: 4.0 mM (●); 2.0 mM (○); and 1.0 mM (▼) *p*NPP. (**b**) Lineweaver–Burk plot for inhibition of PTP1B by *sn*-1,3-dipalmitoyl-2-oleoyl glycerol was analyzed in the presence of different concentrations of sample as follows: 120.19 µM (●), 24.04 µM (○), 4.81 µM (▼), and 0 µM (Δ) *sn*-1,3-dipalmitoyl-2-oleoyl glycerol.

In an attempt to explain the mode of enzymatic inhibition of the most active *sn*-1,3-dipalmitoyl-2-oleoyl, kinetic analyses were performed at different concentrations of the substrate and various inhibitor concentrations. Dixon plots are a graphical method [a plot of 1/enzyme velocity (1/*V*) against inhibitor concentration (I)] for determining the type of enzyme inhibition and *K*_i_ for an enzyme-inhibitor complex. The plots allow easy determination of these attributes, as shown in Figure 4, with the enzymatic kinetic analysis of most active sn-1,3-dipalmitoyl-2-oleoyl. *Sn*-1,3-dipalmitoyl-2-oleoyl showed mixed-type PTP1B inhibition with a *K*_i_ value of 16.36 μM.

### 2.6. Molecular Docking Studies of the Sn-1,3-Dipalmitoyl-2-oleoyl Glycerol Against PTP1B

**Figure 5:**
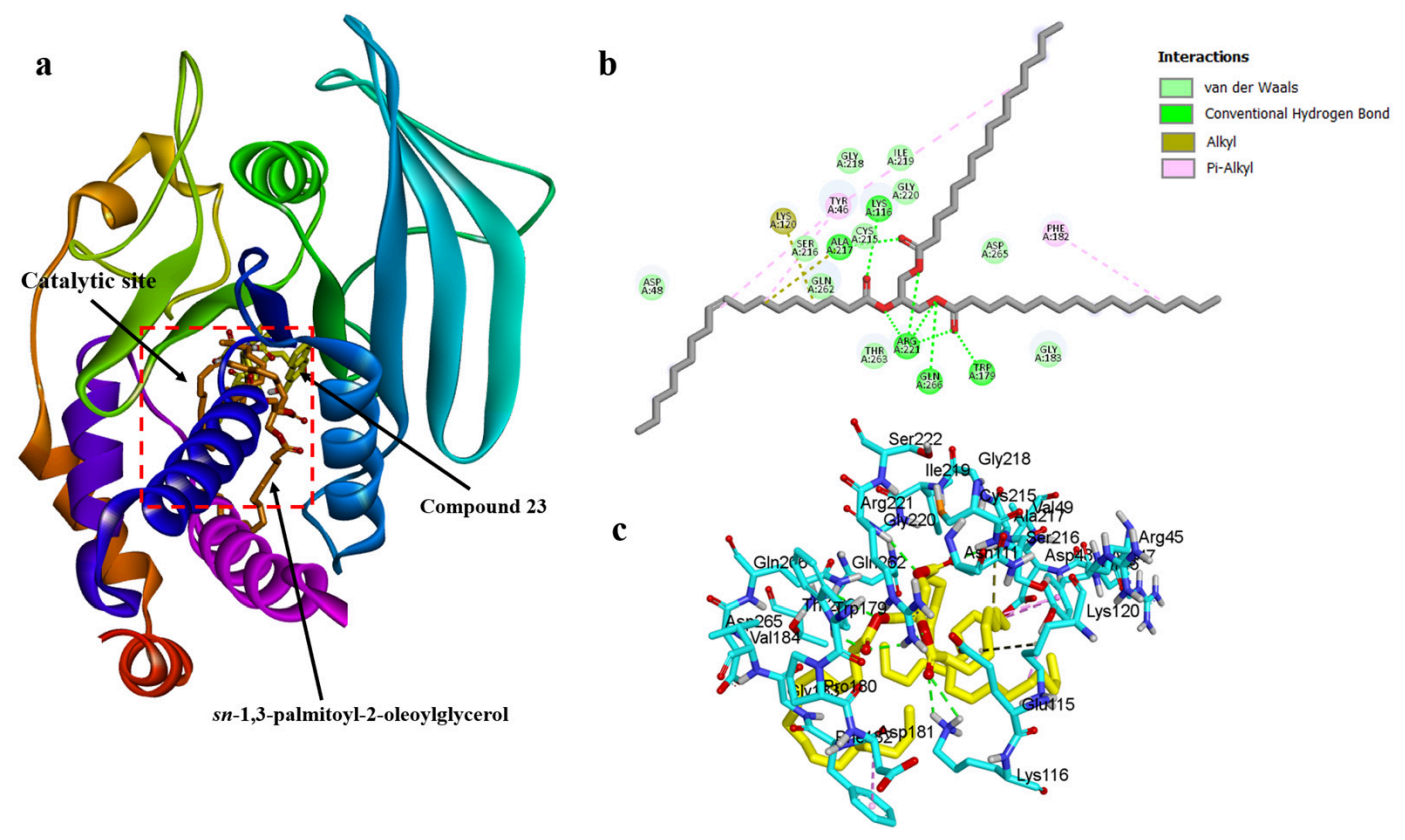
(**a**) Molecular docking of PTP1B inhibition by sn-1,3-dipalmitoyl-2-oleoyl glycerol (brown) and compound 23 (yellow). (**b**) 2D molecular docking model of sn-1,3-dipalmitoyl-2-oleoyl glycerol and (**c**) 3D molecular docking model and major binding interactions of sn-1,3-dipalmitoyl-2-oleoyl glycerol. Interactions are represented in green (conventional hydrogen bonding), lavender (π-alkyl), and lime (alkyl).

A molecular docking simulation in Autodock Vina was used to investigate the binding of *sn*-1,3-dipalmitoyl-2-oleoyl glycerol and compound 23 inside the pocket site of PTP1B. The tested compound *sn*-1,3-dipalmitoyl-2-oleoyl glycerol and compound 23 are shown in brown and yellow, respectively (Figure 5a). Compound 23 was used as co-crystalline ligands for PTP1B, and ligands were extracted from the crystal structures of the proteins. As shown in Figure 5b, 2D interactions occurred between *sn*-1,3-dipalmitoyl-2-oleoyl glycerol and residues within the PTP1B active pocket. As shown in Table 5, *sn*-1,3-dipalmitoyl-2-oleoyl glycerol exhibited −7.4 kcal/mol binding affinity for PTP1B within the catalytic pocket. The probable binding mode of *sn*-1,3-dipalmitoyl-2-oleoyl glycerol within the catalytic site involved conventional hydrogen bonds with Arg221, Gln266, Trp179, Ala217, and Lys116 within the active site of PTP1B (Figure 5b), while Ala217 and Lys120 demonstrated alkyl and Phe182 and Tyr46 π-alkyl interactions. 3D modeling provided better insight into the binding of *sn*-1,3-dipalmitoyl-2-oleoyl glycerol within the active pocket of PTP1B (Figure 5c). Compound 23 exhibited numerous key interactions with PTP1B including conventional hydrogen bonds with Asp48, Arg254, Arg221, Ser216, Gly220, Gly218, Ile219, and Ala217 within the catalytic site of PTP1B (Table 5). Moreover, π-alkyl interactions were observed between compound 23 and Ala27 and Ala217. Some amino acids like Tyr46 and Ala217 exhibited π-sigma interactions. Other interactions included π-sulfur interactions with Met258, π-π stacking with Tyr46, a carbon–hydrogen bond with Tyr46, and π-donor-hydrogen bond interactions with Gly220.

### 2.7. Molecular Dynamics Simulation of sn-1,3-Dipalmitoyl-2-oleoyl Glycerol and PTP1B

**Figure 6:**
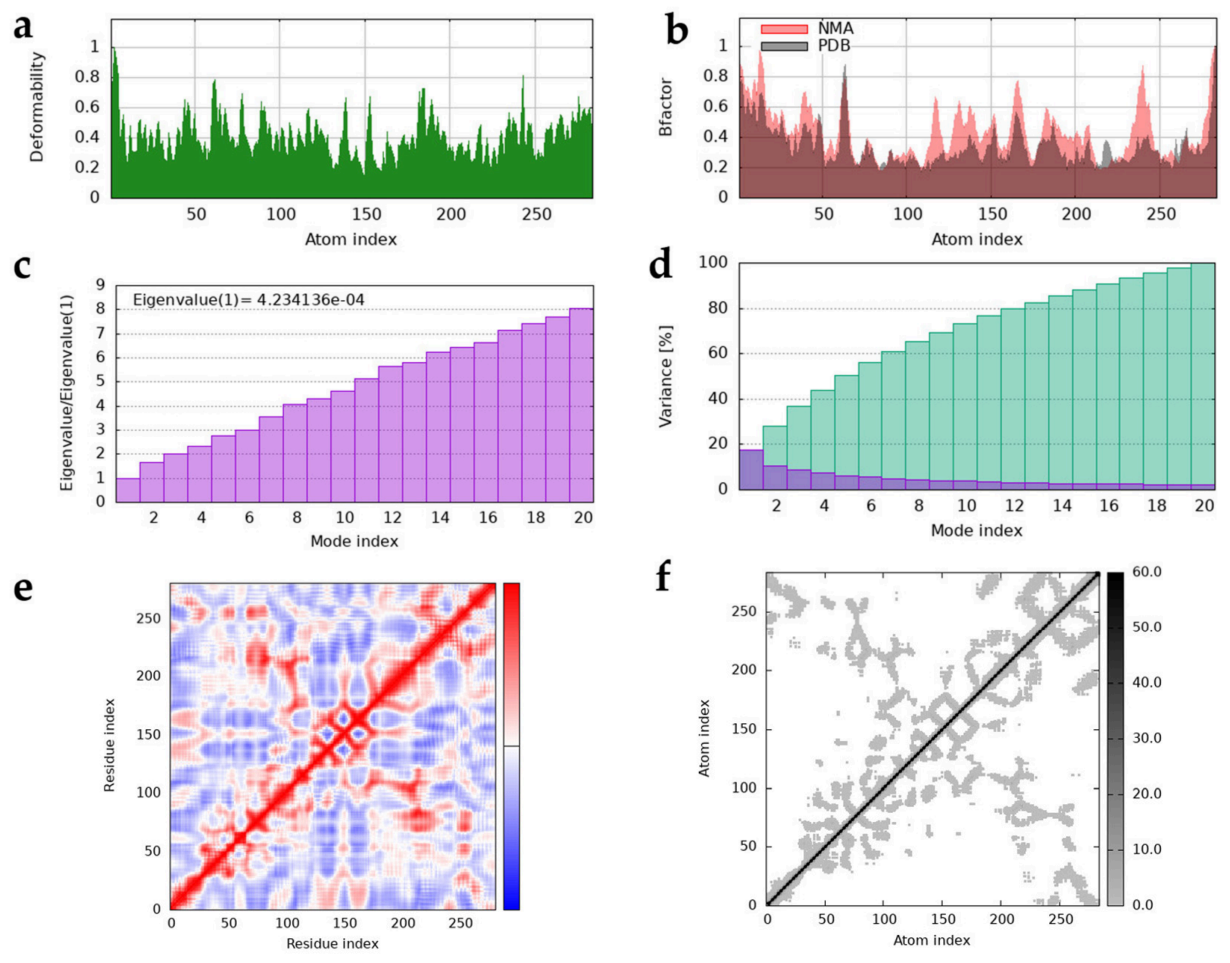
Molecular dynamics simulation analysis of PTP1B in complex with *sn*-1,3-dipalmitoyl-2-oleoyl glycerol.

The deformability plot (Figure 6a) illustrates the protein’s structural flexibility, where peaks denote highly dynamic regions, often associated with loops or binding sites. These fluctuations indicate that specific residues possess notable conformational adaptability, potentially facilitating ligand binding and overall protein functionality. B-factor analysis (Figure 6b) compares theoretical fluctuations from normal mode analysis (NMA) with experimental crystallographic B-factors, revealing alignment in peak positions. This consistency supports the accuracy of the simulation, reinforcing the predicted flexibility pro-file. Notably, elevated fluctuations at the terminal regions suggest inherent structural disorder, while moderate flexibility at the binding site implies a balance between stability and adaptability during inhibitor interaction. Eigenvalue analysis (Figure 6c) reflects the energy required for structural deformation, where the lowest eigenvalue (4.2341 × 10^−4^) corresponds to the most functionally relevant collective motions, indicating a thermodynamically favorable binding process. Variance distribution (Figure 6d) shows that the initial few modes account for the majority of conformational variability, a characteristic feature of biologically relevant protein–ligand interactions. The covariance matrix (Figure 6e) highlights correlated and anti-correlated motions, demonstrating strong correlations within secondary structural elements that contribute to stability, while flexible loop regions exhibit moderate anti-correlations, aiding dynamic ligand accommodation. Additionally, the elastic network model (Figure 6f) visualizes interatomic interactions, revealing densely connected secondary structures that enhance mechanical stability, whereas loop regions display sparse connectivity, supporting an induced-fit binding mechanism.

### 2.8. Molecular Docking Studies of the sn-1,3-Dipalmitoyl-2-oleoyl Glycerol Against α-Glucosidase

**Figure 7:**
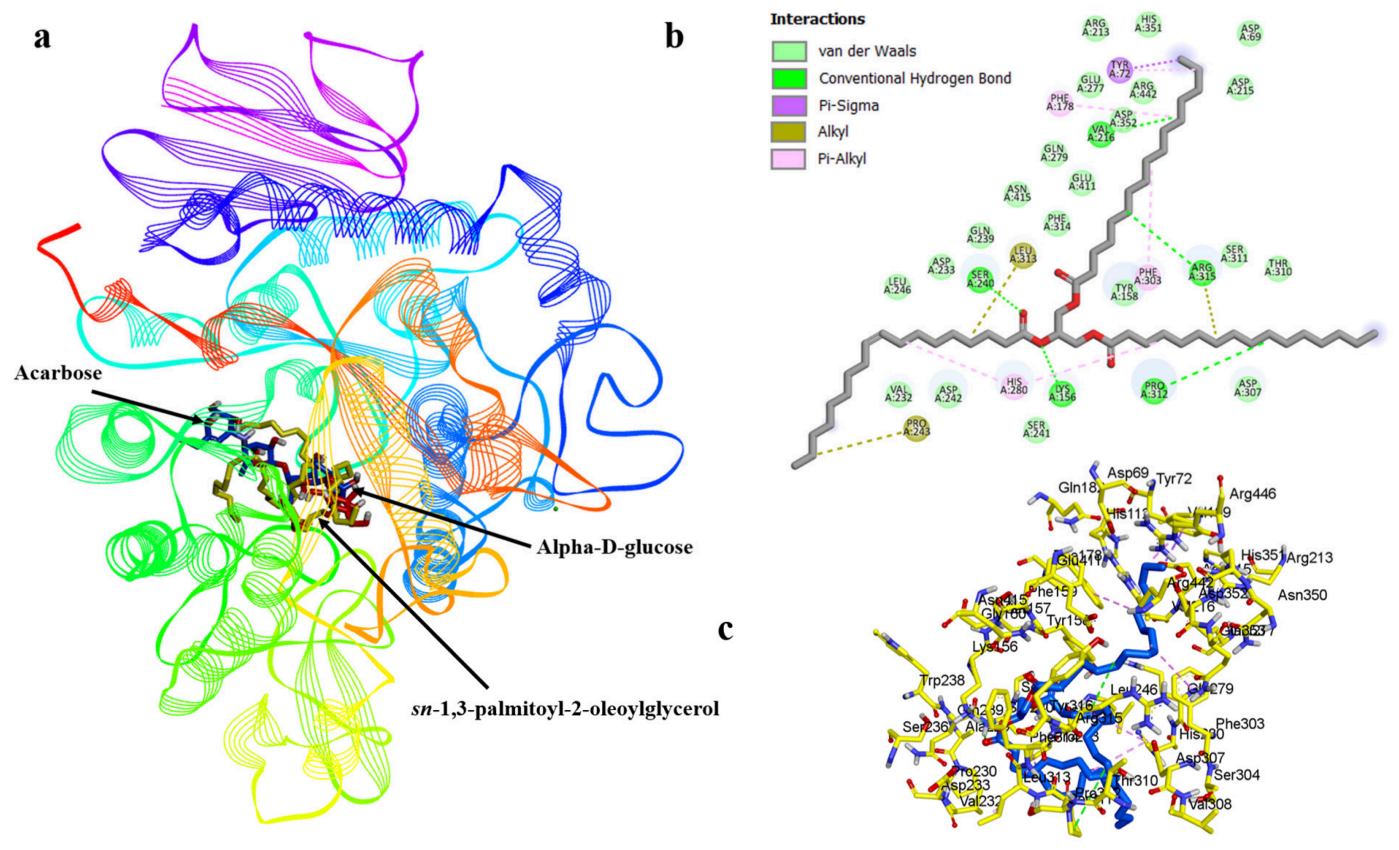
(**a**) Molecular docking of *sn*-1,3-dipalmitoyl-2-oleoyl glycerol (yellow), acarbose (blue), and alpha-D-glucose (red) with α-glucosidase. (**b**) 2D interactions of *sn*-1,3-dipalmitoyl-2-oleoyl glycerol inside the active pocket of α-glucosidase. (**c**) 3D molecular docking model and major binding interactions of *sn*-1,3-dipalmitoyl-2-oleoyl glycerol. Interactions are represented in green (conventional hydrogen bonding), dark lime (alkyl), purple (π-sigma), and lavender (π-alkyl).

Next, the molecular docking of *sn*-1,3-dipalmitoyl-2-oleoyl glycerol to α-glucosidase was performed, and stable ligand–enzyme complexes involving *sn*-1,3-dipalmitoyl-2-oleoyl glycerol (yellow), acarbose (blue) and alpha-D-glucose (red) were generated using Autodock Vina (Figure 7a). As shown in Table 5, *sn*-1,3-dipalmitoyl-2-oleoyl glycerol had a binding affinity of −7.1 kcal/mol to the active pocket of α-glucosidase. *Sn*-1,3-dipalmitoyl-2-oleoyl glycerol exhibited several important interactions with α-glucosidase including hydrogen bonding with Arg315, Lys156, Ser240, Pro312, and Val216 within the active pocket of α-glucosidase (Figure 7b and Table 5), while Gly269 had a carbon–hydrogen bond interaction. Moreover, alkyl interactions were observed between *sn*-1,3-dipalmitoyl-2-oleoyl glycerol and Pro243, Leu313, and Arg315. Some amino acids like His280, Phe303, Phe178, and Tyr72 exhibited π-alkyl interactions, while Tyr72 had a π-sigma interaction. 3D molecular docking model and major binding interactions of *sn*-1,3-dipalmitoyl-2-oleoyl glycerol were observed (Figure 7c).

### 2.9. Molecular Dynamics Simulation of sn-1,3-Dipalmitoyl-2-oleoyl Glycerol and α-Glucosidase

**Figure 8:**
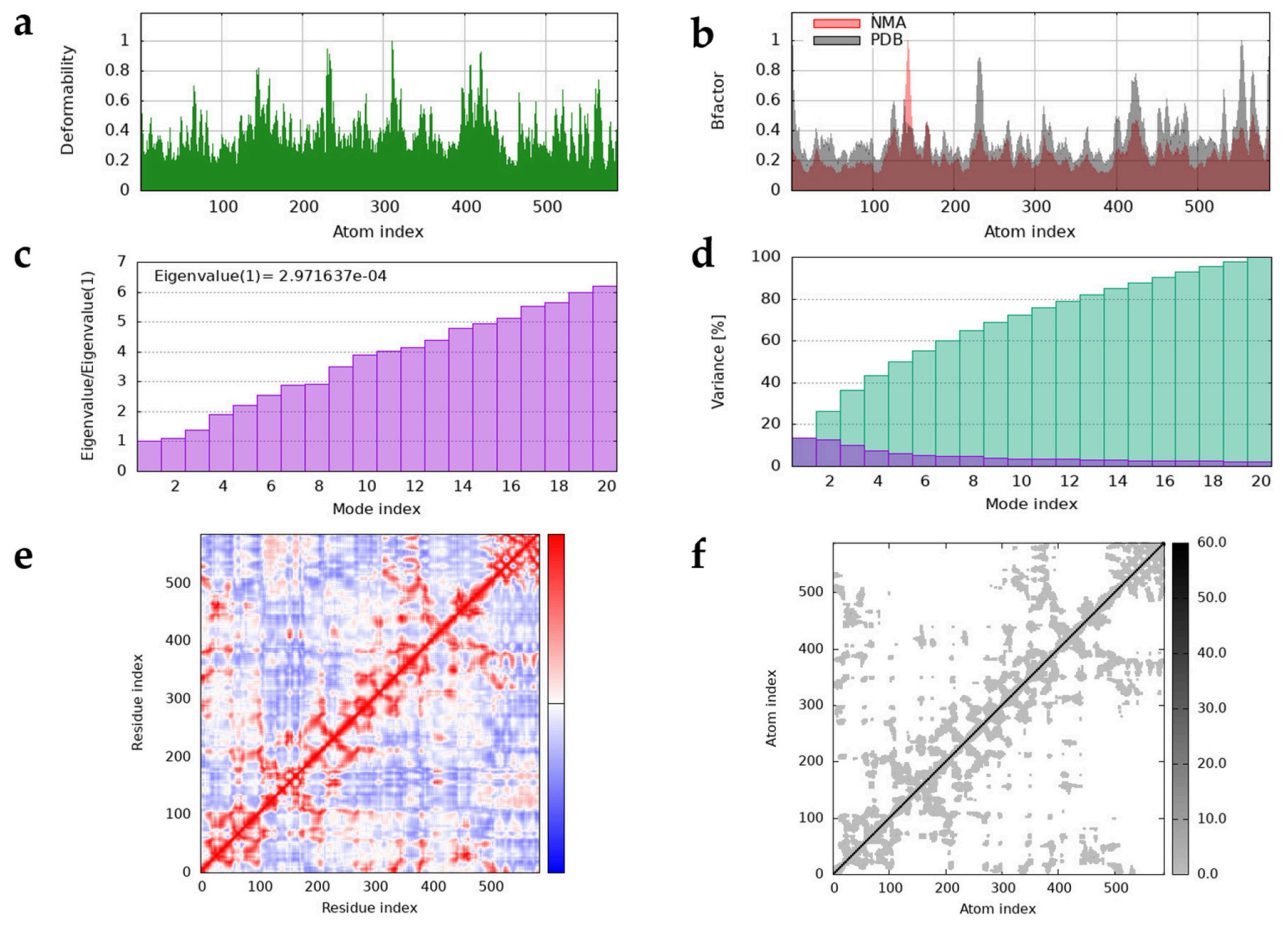
Molecular dynamics simulation analysis of α-glucosidase in complex with sn-1,3-dipalmitoyl-2-oleoylglycerol.

The deformability plot (Figure 8a) depicts the flexibility of individual residues along the protein backbone, where peaks correspond to highly dynamic regions, such as loops and solvent-exposed segments, whereas valleys indicate structurally rigid elements, including α-helices and β-sheets. These fluctuations suggest that certain residues play a pivotal role in overall protein motion, which may influence inhibitor binding and structural integrity. Likewise, the B-factor analysis (Figure 8b) compares theoretical fluctuations from normal mode analysis (NMA) with experimental crystallo-graphic B-factors, showing a strong alignment in fluctuation patterns. Elevated peaks in the NMA-derived B-factors imply increased local flexibility upon inhibitor interaction, which could enhance ligand accommodation and binding stability. The eigenvalue for the primary mode of motion is 2.97 × 10^−4^, reflecting the energy required for conformational transitions (Figure 8c). A lower eigenvalue suggests greater overall flexibility and enhanced global motion in the protein–ligand complex. The variance distribution (Figure 8d) indicates that higher mode indices contribute substantially to structural variation, emphasizing the prominence of functionally significant motions. Furthermore, correlation and mobility patterns, represented by the cross-correlation map (Figure 8e) and atomic covariance matrix (Figure 8f), highlight domain-specific collective movements that facilitate allosteric regulation. The presence of coordinated and opposing residue shifts, depicted in red and blue regions, respectively, underscores the adaptability of the protein structure, reinforcing the crucial role of flexibility in stabilizing the inhibitor-bound conformation.

### 2.10. Inhibitory Effect of sn-1,3-Dipalmitoyl-2-oleoylglycerol and 70% EtOH Soluble Fractions of Skipjack Tuna Heart on ONOO^–^-Mediated Albumin Nitration

**Figure 9:**
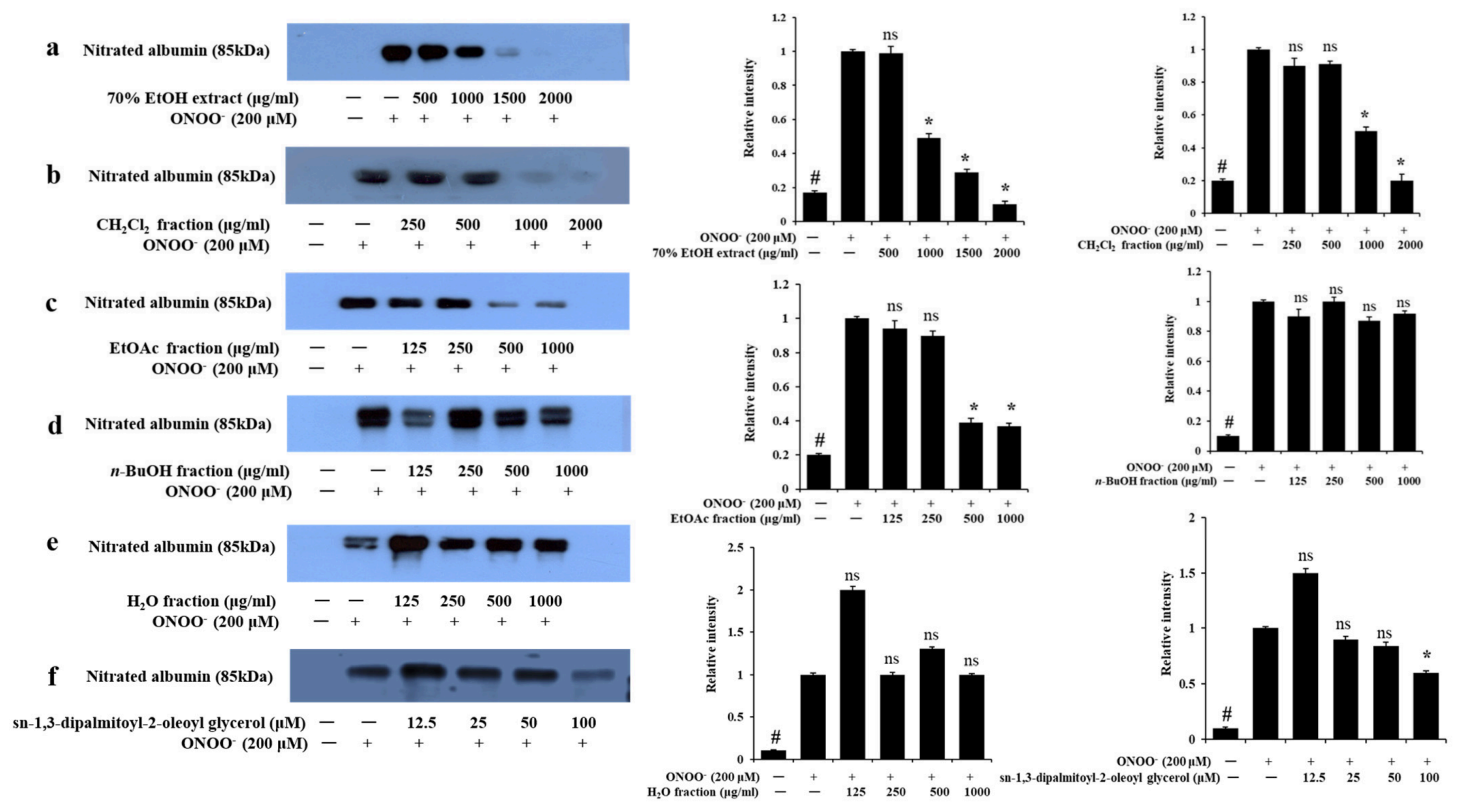
Inhibition of ONOO−-mediated albumin nitration by tuna heart-derived 70% ethanol (EtOH) soluble fractions and compound. Test sample mixtures, bovine serum albumin (BSA), and ONOO− were incubated for 30 min with shaking at 37 °C. The reactant was resolved by electrophoresis in a 10% polyacrylamide gel. (**a**) 70% EtOH tuna heart extract; (**b**) CH2Cl2 fraction; (**c**) EtOAc fraction; (**d**) n-BuOH fraction; (**e**) H2O fraction; and (**f**) sn-1,3-dipalmitoyl-2-oleoyl glycerol were used at the indicated concentrations. Band intensity quantification has been measured using CS Aanlyzer 3.00 (ATTO Corp, Tokyo, Japan). # *p* < 0.05 indicates a significant difference from the untreated normal group, * *p* < 0.05 indicates significant difference from the ONOO− treated control. ns = non-significant. Results were analyzed by ANOVA and Duncan’s test (*p* < 0.05).

To evaluate the inhibitory effect of the 70% EtOH soluble fractions of skipjack tuna heart and most active compound, *sn*-1,3-dipalmitoyl-2-oleoylglycerol, against ONOO^–^-induced nitration of albumin, Western blot analysis was performed using 3-nitrotyrosine antibody. The ONOO^–^-mediated albumin nitration was significantly inhibited by pretreatment with skipjack tuna heart-derived 70% EtOH extract, CH_2_Cl_2_, and EtOAc fractions at varying concentrations (125–2000 μg/mL), as illustrated in Figure 9a–c. Conversely, pretreatment with compound *sn*-1,3-dipalmitoyl-2-oleoylglycerol at a concentration of 100 μM moderately inhibited ONOO^–^-mediated albumin nitration (Figure 9f). Nevertheless, pretreatment with *n*-BuOH and H_2_O fractions at varying doses (125–1000 μg/mL) did not inhibit ONOO−mediated albumin nitration (Figure 9d,e).

## 3. Materials and Methods

### 3.1. Extraction, Fractionation and Isolation of Active Compounds from Tuna Heart

Tuna heart powder was purchased from Dongwon F&B Co. (Seongnam, Republic of Korea). Two kilograms of the powder was refluxed with 70% EtOH three times for 3 h each time. The total filtrate was concentrated to dryness in vacuo at 40 °C to acquire the EtOH extract. The extract (445.84 g) was suspended in distilled water and partitioned with dichloromethane (CH_2_Cl_2_), ethyl acetate (EtOAc), and *n*-butanol (*n*-BuOH) to yield the CH_2_Cl_2_ (236.2 g), EtOAc (5.12 g), and *n*-BuOH (16.4 g) fractions, respectively, as well as an aqueous residue (126.58 g) (Figure 1). The high yield CH_2_Cl_2_ fraction was subjected to column chromatography over a silica (Si)-gel column using a *n*-hexane-EtOAc gradient to obtain 10 subfractions (F-1 to F-10). Among these, F-4 (3.03 g) was chromatographed over a Si-gel column using hexane-EtOAc to yield five subfractions (F-4-1 to F-4-5). Subfraction F-4-3 (1.41 g) was chromatographed over a Si-gel column using hexane-EtOAc to obtain seven subfractions (F-4-3-1 to F-4-3-7). Subfraction F-4-3-2 (205 mg) was repeatedly chromatographed over a Si-gel column using hexane-EtOAc to obtain cholesteryl myristate (20 mg). Subtraction F-4-3-5 was repeatedly chromatographed over a Si-gel column with CH_2_Cl_2_ to obtain *sn*-1,3-dipalmitoyl-2-oleoylglycerol (140 mg). F-3 (0.83 g) was chromatographed over a Si-gel column using hexane-acetone to give seven subfractions (F-3-1 to F-3-7). Subfraction F-3-2 (0.58 g) was chromatographed over a Si-gel column using hexane-EtOAc to give four subfractions (F-3-4-1 to F-3-4-4). F-3-4-2 and F-3-4-4 were recombined and recrystallized with CH_2_Cl_2_ to yield cholesterol (300 mg). Cholesterol myristate, *sn*-1,3-dipalmitoyl-2-oleoylglycerol and cholesterol were identified by spectroscopic analysis, including ^1^H- and ^13^C nuclear magnetic resonance (NMR), by comparison with published spectral data, and by thin layer chromatography (TLC) analysis [23,24,25]. All isolated compounds were analyzed and identified using spectroscopic methods, including ^1^H, ^13^C NMR, and mass data, which are provided in the Supplementary Information Section.

### 3.2. PTP1B Inhibition Assay

PTP1B inhibitory activity was evaluated using *p*-nitrophenylphosphate (*p*NPP) as described previously [7]. Ursolic acid was used as a positive control.

### 3.3. α-Glucosidase Inhibition Assay

The assay was performed spectrophotometrically as previously detailed [7] using acarbose as a positive control.

### 3.4. Inhibition of ONOO−-Mediated Protein Tyrosine Nitration

ONOO−-mediated protein tyrosine nitration was evaluated with minor modifications by using in our previously reported method [49].

### 3.5. Kinetic Parameters of sn-1,3-Dipalmitoyl-2-oleoylglycerol in PTP1B Inhibition

To determine the kinetic mechanism, Lineweaver–Burk and Dixon plots of kinetic activity were used. PTP1B inhibition mode was determined at various concentrations of *p*NPP substrate (0.25, 0.5, and 1.0 mM) in the absence or presence (0, 4.81, 24.04, and 120.19 µM) of *sn*-1,3-dipalmitoyl-2-oleoylglycerol. Inhibition of *sn*-1,3-dipalmitoyl-2-oleoylglycerol was evaluated by monitoring the effects of different concentrations of the substrates in Dixon plots (single reciprocal plot). The enzymatic procedures consisted of the aforementioned PTP1B assay. The inhibition constant (*K_i_*) were determined by interpretation of the Dixon plots, where the value of the *x*-axis implied -*K_i_*. 

### 3.6. Molecular Docking Simulation of sn-1,3-Dipalmitoyl-2-oleoyl Glycerol with PTP1B and α-Glucosidase

To investigate the binding of *sn*-1,3-dipalmitoyl-2-oleoyl glycerol inside active receptor pockets, the protein structures for PTP1B (PDB ID: 1NNY) [50] and α-glucosidase (PDB ID: 3A4A) [51] were downloaded from the Protein Data Bank (pdb). These protein structures were originally obtained using an X-ray diffraction method. Reported heteroatom compounds were removed, and proteins were regarded to be ligand-free. Water molecules were also removed from protein structures for the docking simulation using Accelrys Discovery Studio 4.1 (DS 4.1) (http://www.accelrys.com; accessed on 1 September 2022; Accelrys, Inc. San Diego, CA, USA). Polar hydrogen atoms were added to the protein using the automated docking tool Autodock 4.2.6. Docking studies of *sn*-1,3-dipalmitoyl-2-oleoyl glycerol, acarbose, and co-crystalline ligands were performed without modifying the default parameters. For the docking studies, the binding areas of compound 23 and alpha-D-glucose were considered the most likely regions for docking with the proteins. 2D structures of compounds were drawn with MarvinSketch (www.chemaxon.com accessed date 1 July 2022; Chemaxon, Life Sciences, Informatics, Cheminformatics, Budapest, Hungary) and converted into 3D pdb format using DS 4.1. A grid box size of 60 × 60 × 60 points with a spacing of 1.0 Å between the grid points was implemented to cover almost all favorable protein binding sites. X, Y, Z centers were as follows: PTP1B (31.15, 28.65, and 21.49, respectively) and α-glucosidase (21.28, −0.75, and 18.63). In the docking studies, the selected ligands were examined to find qualified binding poses for each compound. Binding interactions of PTP1B and α-glucosidase residues and corresponding binding affinity scores were regarded as the best molecular interactions. Results were analyzed using Discovery Studio Visualizer (Accelrys Software Inc. 2013, San Diego, CA, USA). Two-dimensional figures of compound–protein interactions were created using Discovery Studio Visualizer (Accelrys Software Inc. 2013, San Diego, CA, USA). 

### 3.7. Molecular Dynamics Simulation

Molecular dynamics simulations (MDSs) were performed using the iMODS web server to evaluate the conformational flexibility and stability of *sn*-1,3-dipalmitoyl-2-oleoyl in complex with protein tyrosine phosphatase 1B (PTP1B) and *α*-glucosidase. The iMODS platform applies normal mode analysis (NMA) in internal coordinates, facilitating the examination of large-scale macromolecular movements while preserving stereochemical integrity. Hessian matrix-based computations provided information regarding atomic displacement patterns, deformation energy, and covariance mapping of residue fluctuations [52].

## 4. Conclusions

In summary, the anti-diabetic properties of various skipjack tuna heart fractions were examined in this study. We identified three effective compounds in the CH_2_Cl_2_ fraction. The effectiveness of tuna heart extract and the isolated component *sn*-1,3-dipalmitoyl-2-oleoyl in the inhibition of PTP1B and α-glucosidase was observed. *Sn*-1,3-dipalmitoyl-2-oleoyl showed mixed-type of inhibition in PTP1B kinetic analyses, and the molecular docking data suggested that this compound may be a good candidate therapeutic agent to treat and prevent DM by targeting the inhibition of PTP1B. It was found that 70% EtOH soluble fractions of skipjack tuna heart and most active compound, *sn*-1,3-dipalmitoyl-2-oleoylglycerol, also effectively suppressed ONOO−-mediated albumin nitration. The results demonstrate the anti-diabetic effect of skipjack tuna heart extract, which may be useful in the development of multiple therapies for treatment of DM and its associated diseases. Moreover, the present study provides useful information for increasing the commercial value of skipjack tuna heart extract as a multifunctional ingredient and suggests new ways to increase its recognition as a natural source of anti-diabetic agents that are capable of preventing DM and associated complications.

## Figures and Tables

**Table 1:** Anti-diabetic properties of various solvent fractions from tuna 70% EtOH extract.

Sample	IC_50_ (µg/mL)	
	PTP1B	α-Glucosidase
CH_2_Cl_2_ fraction	33.53 ± 0.33	609.51 ± 20.89
EtOAc fraction	>1000	612.55 ± 23.38
*n*-BuOH fraction	884.73 ± 13.11	>1000
H_2_O fraction	758.99 ± 47.39	>1000
Ursolic acid ^a^	4.95 ± 0.05	
Acarbose ^b^		170.02 ± 1.64

The 50% inhibition concentrations (µg/mL) were calculated from a log dose inhibition curve and expressed as the mean ± S.E.M. of triplicate experiment. ^a,b^ Positive control used in different assays.

**Table 2:** Effect of various subfractions derived from the CH_2_Cl_2_ fraction of tuna heart 70% EtOH extract on the PTP1B and a-glucosidase.

Samples	Yield (g)	500 μg/mL (Inhibition %, Mean ± SEM) ^a^	
		PTP1B	α-Glucosidase
F-1	0.72	93.17 ± 0.13	9.27 ± 0.80
F-2	0.05	68.02 ± 2.08	34.34 ± 0.57
F-3	0.83	116.65 ± 0.33	82.01 ± 0.38
F-4	3.03	84.59 ± 6.20	80.76 ± 1.56
F-5	0.41	99.14 ± 0.05	95.26 ± 0.52
F-6	0.40	98.17 ± 0.01	67.00 ± 0.50
F-7	0.34	26.69 ± 1.58	31.72 ± 0.10
F-8	0.21	81.53 ± 0.02	24.09 ± 0.84
F-9	0.15	101.33 ± 1.61	58.33 ± 2.15
F-10	12.79	78.53 ± 0.46	6.75 ± 0.79
Ursolic acid ^b^		69.52 ± 1.95	
Acarbose ^c^			60.76 ± 0.37

^a^ Values are expressed as the mean ± SEM of triplicate experiments. ^b^ Ursolic acid (10 µg/mL) and ^c^ acarbose (250 µg/mL) were used as a positive control.

**Table 3:** PTP1B and α-glucosidase inhibitory activities of compounds from skipjack tuna heart.

Compounds	PTP1B	α-Glucosidase
	IC_50_ (µM) ^a^	
Cholesterol	518.13 ± 2.56	647.67 ± 3.64
Cholesterylmyristate	335.57 ± 1.24	419.46 ± 2.10
*sn*-1,3-dipalmitoyl-2-oleoyl glycerol	31.01 ± 0.39	163.31 ± 1.15
Ursolic acid ^b^	12.58 ± 0.08	
Acarbose ^c^		188.14 ± 2.39

^a^ The 50% inhibition concentration (µM) was calculated from a log dose inhibition curve and is expressed as the mean ± SEM from triplicate experiments. ^b,c^ Used as positive controls.

**Table 4:** PTP1B inhibitory activity of components of *sn*-1,3-dipalmitoyl-2-oleoyl glycerol.

Compounds	IC_50_^a^
Glycerol	>300
Palmitic acid	204.58 ± 15.05
Oleic acid	64.19 ± 0.22
*sn*-1,3-dipalmitoyl-2-oleoyl glycerol	31.01 ± 0.39
Ursolic acid ^b^	8.48 ± 0.45

^a^ The 50% inhibitory concentration (IC_50_) value (μM) was calculated from a log dose inhibition curve and expressed as mean ± SEM of triplicate experiments. ^b^ Positive control.

**Table 5:** Binding energies and interactions of *sn*-1,3-dipalmitoyl-2-oleoyl glycerol and cognate ligands docked with PTP1B and α-glucosidase using the Autodock Vina docking program and visualization by Discovery Studio Visualizer.

Target Enzymes	PDB ID	Ligands	Binding Energies (Kcal/mol) ^a^	Hydrogen Bond Interacting Residues and Bonding Distance	Hydrophobic Interactions
PTP1B	1NNY	*sn*-1,3-dipalmitoyl-2-oleoyl glycerol ^b^	−7.4	Arg221 (2.30, 2.53, 1.98, and 2.20 Å), Gln266 (2.82 Å), Trp179 (2.07 Å), Ala217 (2.11 Å) and Lys116 (2.68 Å)	Ala217 (alkyl, 4.12 Å), Lys120 (alkyl, 5.03 Å), Phe182 (π-alkyl, 5.46 Å), Tyr46 (π-alkyl, 5.25, 3.88, and 4.89 Å)
		Compound 23 (Catalytic inhibitor) ^c^	−8.8	Arg221 (2.80 and 3.05 Å), Ile219 (3.01 Å), Arg254 (2.86 Å), Gly218 (3.39 Å), Asp48 (2.82 and 2.64 Å), Ser216 (3.14 Å), Gly220 (2.72 Å), Ala217 (2.69 Å)	Tyr46 (π-sigma, 3.57 Å), Ala217 (π-sigma, 3.87 Å), Gly220 (π-donor-hydrogen bond, 3.88Å), Ala27 (π-alkyl, 5.09 Å), Ala217 (π-alkyl, 5.36 Å), Met258 (π-sulfur, 5.59 and 5.91 Å), Tyr46 (π-π stacked 5.21 Å), Tyr46 (carbon–hydrogen bond, 3.94 Å)
α-Glucosidase	3A4A	*sn*-1,3-dipalmitoyl-2-oleoyl glycerol	−7.1	Arg315 (2.46 Å), Lys156 (2.07 Å), Ser240 (2.13 Å), Pro312 (2.27 Å), Val216 (2.10 Å)	Tyr72 (π-sigma, 3.65 Å), Pro243 (alkyl, 4.21 Å), Leu313 (alkyl, 5.37 Å), Arg315 (alkyl, 4.80 Å), His280 (π-alkyl, 4.75 and 5.15 Å), Phe303 (π-alkyl, 5.05 Å), Phe178 (π-alkyl, 5.26 Å), Tyr72 (π-alkyl, 4.24 Å)
		Acarbose	−8.3	Asp352 (2.38 Å), Asp215 (2.90 Å), Arg442 (2.31 Å), Gln279 (3.02 Å), Pro312 (3.08 Å), Ser240 (2.90 Å), Tyr158 (2.73 Å)	Pro312 (carbon–hydrogen bond, 2.68 Å), His280 (π-sigma, 3.93 Å), Glu411 (unfavorable accepter-accepter, 2.93 Å)
		Alpha-D-glucose	−6.9	Asp69 (2.63 Å), Arg442 (2.78 Å), Arg213 (2.89 Å), Asp352 (2.67 and 2.52Å), Asp215 (2.88 Å), Glu277 (2.75 Å), His112 (2.77 Å), His351 (3.01 and 3.01 Å)	Tyr72 (π-donor hydrogen bond 3.93 Å), Asp69 (carbon–hydrogen bond, 3.41 Å)

^a^ Estimated binding free energy of the ligand-receptor complex. ^b^ sn-1,3-dipalmitoyl-2-oleoyl glycerol. ^c^ Compound 23 (3-({5-[(N-acetyl-3-{4-[(carboxycarbonyl)(2-carboxyphenyl)amino]-1-naphthyl}-L-alanyl)amino]pentyl}oxy)-2-naphthoic acid), acarbose, and Alpha-D-glucose were used as positive ligands.

## Data Availability

The original contributions presented in this study are included in the article. Further inquiries can be directed to the corresponding authors.

## Supplementary Materials

The following supporting information can be downloaded at: https://www.mdpi.com/article/10.3390/ijms27156914/s1.
