# Supplementary material for: Activity-Guided Isolation of sn-1,3-Dipalmitoyl-2-oleoylglycerol as Protein Tyrosine Phosphatase 1B and α-Glucosidase Inhibitor from Skipjack Tuna (Katsuwonus pelamis)
Source: Int J Mol Sci. 2026 Aug 1;27(15):6914. doi: 10.3390/ijms27156914 (PMC13466518; doi:10.3390/ijms27156914)
Supplement: Supplementary file 1 [file ijms-27-06914-s001.zip › ijms-4449445-supplementary.pdf]

Activity-Guided Isolation of *sn*-1,3-Dipalmitoyl-2-oleoylglycerol as Protein Tyrosine Phosphatase 1B and *α*-Glucosidase Inhibitor from Skipjack Tuna (*Katsuwonus pelamis*)

Md Yousof Ali [1,2], Da Hye Kim [2], Hee Jin Jung [2], Taek Jeong Nam [2] and Jae Sue Choi [2,*]

[1] Department of Clinical Neurosciences, Hotchkiss Brain Institute and Alberta Children's Hospital Research Institute, Cumming School of Medicine, University of Calgary, Calgary, AB T2N 4N1, Canada; mdyousof.ali@ucalgary.ca
[2] Department of Food Science and Nutrition, Pukyong National University, Busan 608-737, Republic of Korea; md2253@naver.com (D.H.K.); king2046@hanmail.net (H.J.J.); namtj@pknu.ac.kr (T.J.N.)
* Correspondence: choijs@pknu.ac.kr; Tel.: +82-51-629-5845

^{1}H-NMR (400 MHz, $CDCl_3$) δ: 5.37 (1H, d, *J* = 4.8 Hz, H-6), 4.61 (1H, m, H-3), 2.32 (1H, brs, H4), 2.30 (1H, brs, H4), 2.26 (2H, t, *J* = 7.5 Hz, H2'), 1.25 (28H, s, $(CH_2)_x$ chain), 1.02 (3H, s, H19, CH_3), 0.91 (3H, d, *J* = 6.6 Hz, H21, CH_3), 0.88 (3H, t, *J* = 6.9 Hz, H14', CH_3), 0.87 (3H, d, *J* = 6.6 Hz, H26, CH_3), 0.86 (3H, d, *J* = 6.6 Hz, H27, CH_3), 0.68 (3H, s, H-18, CH_3)

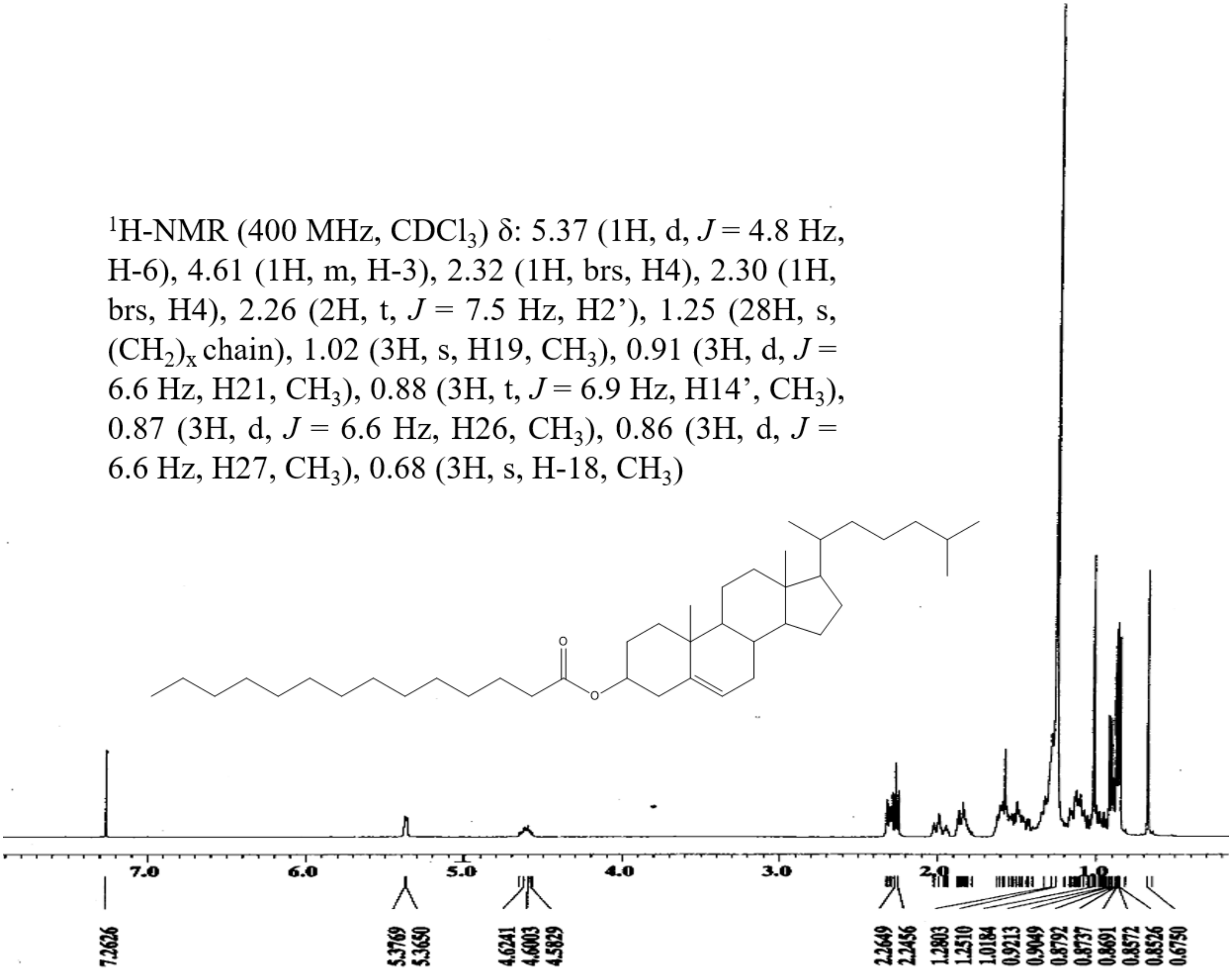

^{1}H NMR spectrum of cholesteryl myristate in $CDCl_3$

^{13}C-NMR (100 MHz, $CDCl_3$) δ : 173.35 (C-1'), 139.68 (C-5), 122.57 (C-6), 73.64 (C-3), 56.64 (C-14), 56.07 (C-17), 49.97 (C-9), 42.27 (C-13), 39.68 (C-12), 39.48 (C-24), 38.12 (C-4), 36.96 (C-1), 36.56 (C-10), 36.14 (C-22), 35.78 (C-20), 34.71 (C-2'), 31.92 (C-12'), 31.88 (C-7), 31.81 (C-8), 29.69 (C-6', 7', 8'), 29.58 (C-4'), 29.45 (C-5'), 29.37 (C-9'), 29.25 (C-10'), 29.10 (C-11'), 28.21 (C-16), 27.99 (C-25), 27.78 (C-2), 25.05 (C-3'), 24.26 (C-15), 23.80 (C-23), 22.82 (C-27), 22.69 (C-13'), 22.55 (C-26), 20.99 (C-11), 19.31 (C-19), 18.69 (C-21), 14.14 (C-14'), 11.83 (C-18)

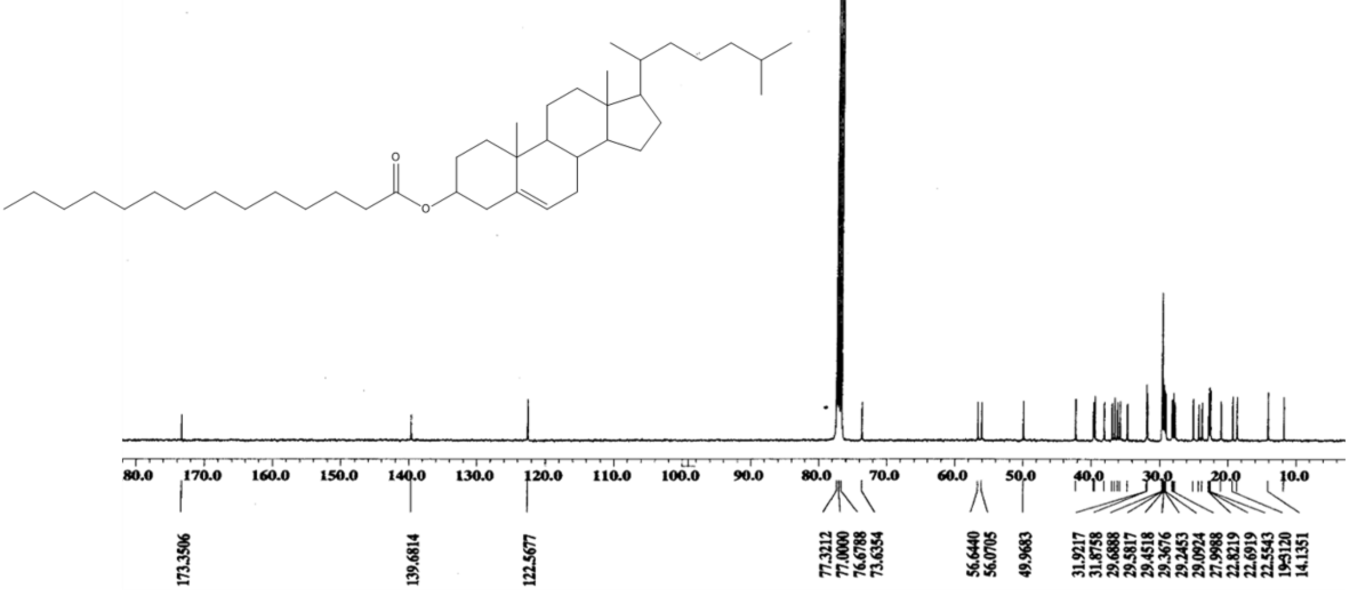

^{13}C NMR spectrum of cholesteryl myristate in $CDCl_3$

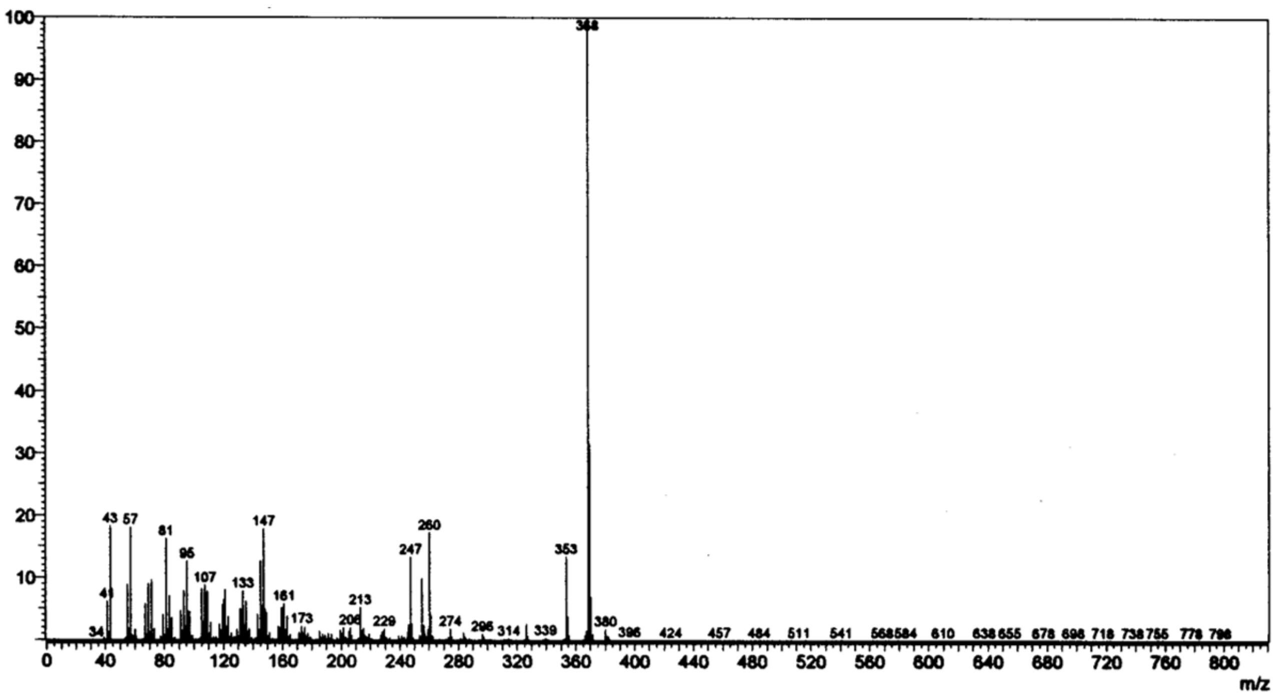

EI-MS spectrum of cholesteryl myrisate

^{1}H-NMR (400 MHz, $CDCl_3$) δ: 4.30 (2H, dd, J = 4.4, 11.92 Hz, glyceryl CH_2O), 4.15 (2H, dd, J = 5.9, 12.08 Hz, glyceryl CH_2O), 5.29 (1H, m, glyceryl CHO), 2.31 (6H, t, J = 7.7 Hz, CH_2), 1.60 (6H, S, CH_2), 5.34, 5.31 (m, olefinic H), 2.00 (allylic, CH_2), 1.25-1.30 (CH_2), 0.87 (3H, t, J = 6.9 Hz, CH_3)

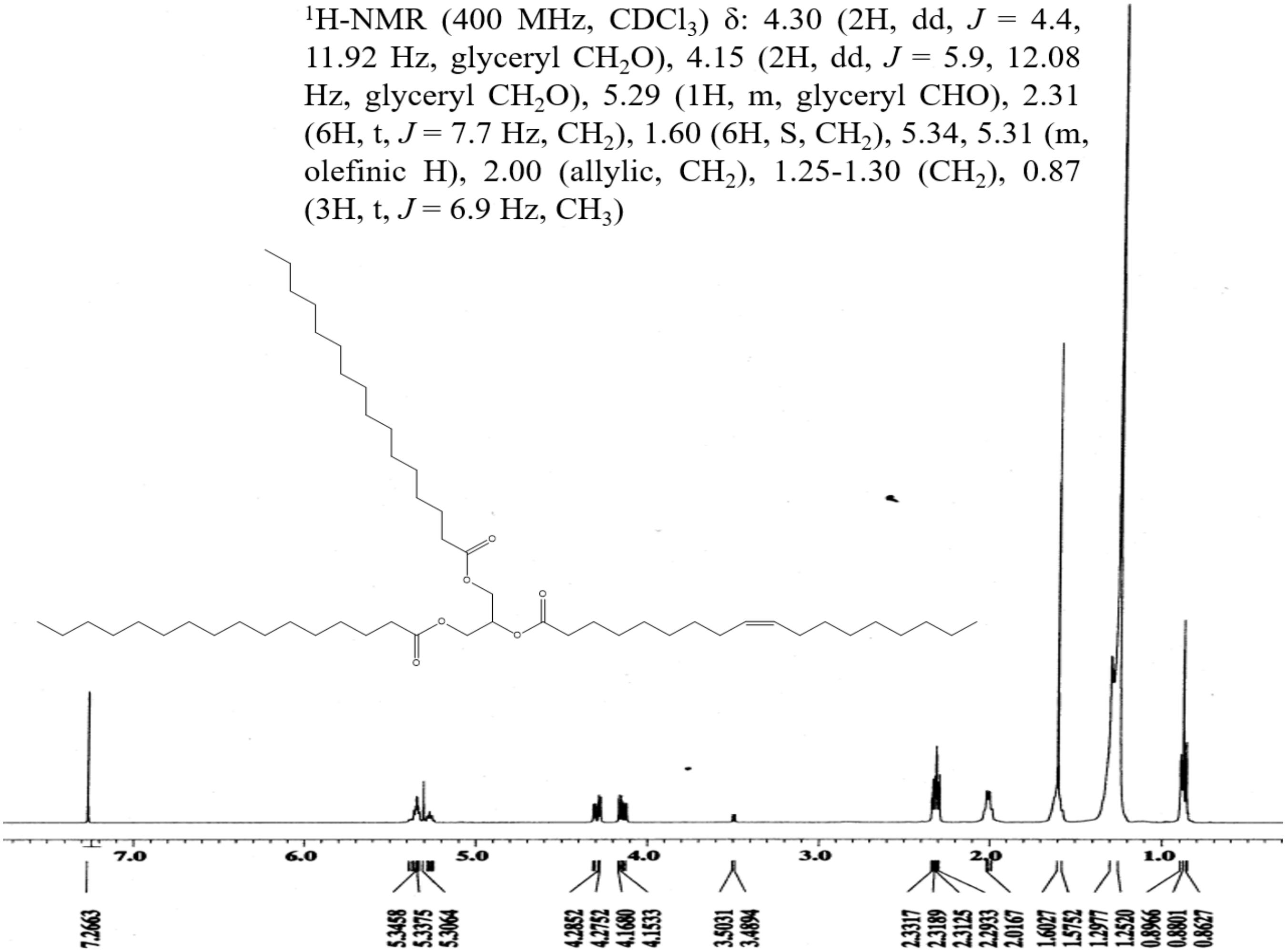

^{1}H NMR spectrum of *sn*-1,3-dipalmitoyl-2-oleoylglycerol in $CDCl_3$

^{13}C-NMR (100 MHz, $CDCl_3$) δ : 62.11 (glyceryl CH_2), 68.84 (glyceryl CH), 173.35 (C=O), 172.93 (C=O), 34.05, 34.23 (C-2), 24.87 (C-3), 29.13 (C-4), 29.33, 29.38, 29.52, 29.72, 22.70 (CH_2), 130.02 (C-10), 129.71 (C-9), 31.93 (CH_2), 14.15 (CH_3)

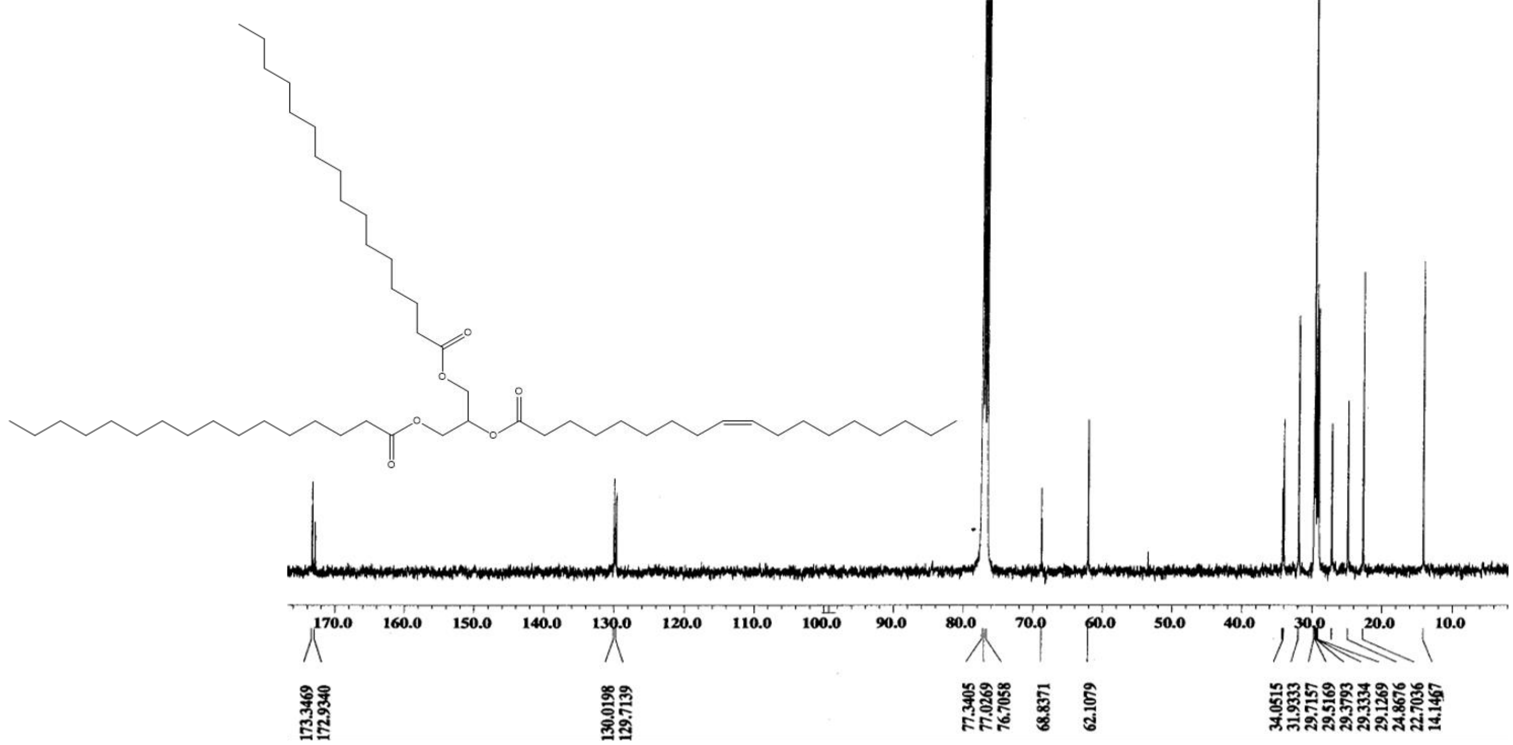

^{13}C NMR spectrum of *sn*-1,3-dipalmitoyl-2-oleoyl glycerol in $CDCl_3$

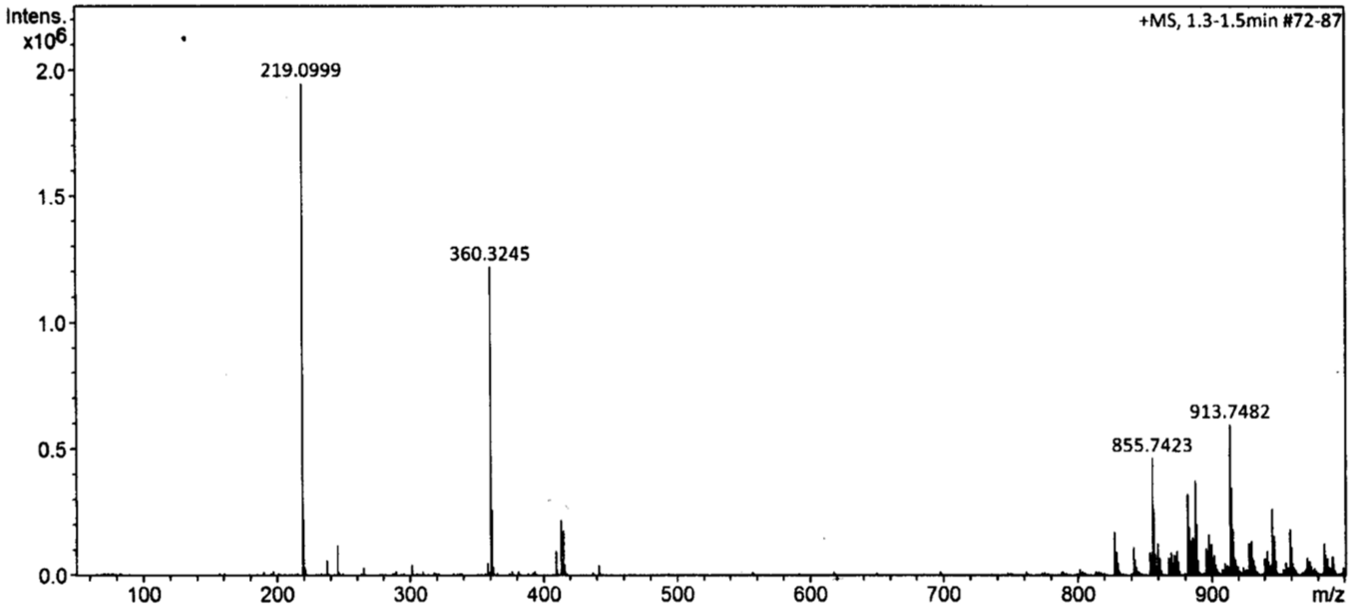

ESI-MS spectrum of *sn*-1,3-dipalmitoyl-2-oleoyl glycerol

ESI-MS *m/z* 855.7423 $[M+Na]^+$ (calcd. 855.7412 for $C_{53}H_{100}NaO_6$)

^{1}H-NMR (400 MHz, $CDCl_3$) δ: 5.35 (1H, d, *J* = 5.5 Hz, H-6), 3.53 (1H, m, H-3), 2.31 (2H, brs, H-4), 2.00 (2H, H-7), 1.84 (6H, m, H-15, H-16, H-20, H-25), 1.58-1.30 (14H, m, H-1, H-2, H-8, H-9, H-11, H-12, H-14, H-17), 1.25 (6H, CH_2, H-22, H-23, H-24), 1.10 (3H, CH_3, H-19), 1.00 (3H, CH_3, H-21), 0.90 (3H, d, *J* = Hz, CH_3, H-27), 0.86 (3H, dd, *J* = Hz, CH_3, H-26), 0.68 (3H, CH_3, H-18)

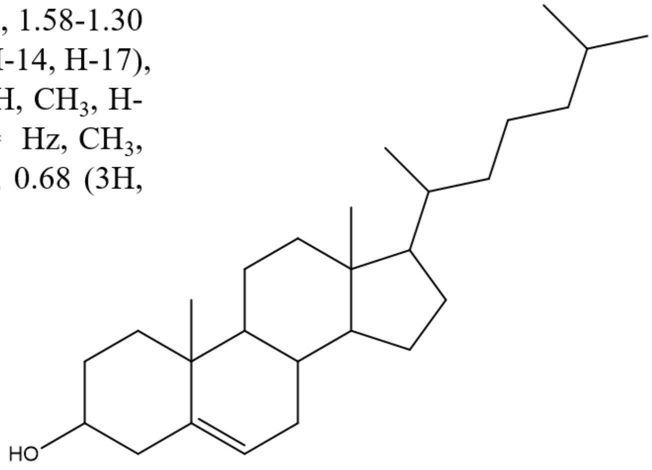

cholesterol

^{13}C-NMR (100 MHz, $CDCl_3$) δ : 140.76 (C-5), 121.73 (C-6), 71.81 (C-3), 56.77 (C-14), 56.15 (C-17), 50.13 (C-9), 42.32 (C-13), 42.30 (C-4), 39.77 (C-12), 39.53 (C-24), 37.27 (C-1), 36.51 (C-10), 36.19 (C-22), 35.80 (C-20), 31.91 (C-7, C-8), 31.66 (C-2), 28.24 (C-16), 28.03 (C-25), 24.30 (C-15), 23.84 (C-23), 22.70 (C-27), 22.57 (C-26), 21.09 (C-11), 19.41 (C-19), 18.72 (C-21), 11.86 (C-18)
